# Ameliorating loneliness through Cognitive Stimulation Therapy and the role of baseline loneliness in predicting cognitive, behavioural and psychological benefits in people with dementia

**DOI:** 10.3389/fpsyg.2025.1656626

**Published:** 2025-10-29

**Authors:** Riccardo Domenicucci, Elena Carbone, Federica Piras, Erika Borella

**Affiliations:** ^1^Department of General Psychology, University of Padova, Padova, Italy; ^2^Laboratory of Neuropsychiatry, Department of Clinical Neuroscience and Neurorehabilitation, Santa Lucia Foundation IRCCS, Rome, Italy

**Keywords:** Cognitive Stimulation Therapy, psychosocial intervention, person-centred care, dementia, loneliness, quality of life, individual differences

## Abstract

**Introduction:**

We examined whether Cognitive Stimulation Therapy (CST) would ameliorate loneliness and its social and emotional components in the short and long-term among people with mild-to-moderate dementia. The role of loneliness and its dimensions, as individual characteristics, in explaining short- and long-term cognitive, behavioural, and psychological CST benefits was also assessed.

**Materials and methods:**

People with dementia, either receiving the Italian adaptation of CST (CST group: *n* = 68) or treatment-as-usual (control group: *n* = 47), were selected from a previous multicenter controlled clinical trial on CST efficacy. They completed the de Jong Loneliness Scale along with measures of general cognitive functioning, language, mood, behaviour, and quality of life before CST intervention, immediately after the treatment, and 3 months later.

**Results:**

A specific short-term reduction in emotional loneliness was found for the CST group compared to controls but it was no longer observable at follow-up. Baseline total loneliness helped explain short-term improvements in depressive symptoms and short- and long-term benefits in quality of life. Specifically, lower baseline social loneliness accounted for short-term decrease in depressive symptoms, whereas higher baseline emotional loneliness explained short- and long-term benefits in quality of life.

**Conclusion:**

CST can reduce emotional loneliness in PwD, albeit in the short-term. Moreover, individual dispositions in terms of social and emotional loneliness seem to have a modest influence on CST’s benefits in mood and quality of life. Loneliness in PwD should be systematically addressed in psychosocial interventions, also to direct individuals who are more predisposed to derive benefits from approaches such as CST.

## Introduction

Loneliness is described as the subjective, negative feeling arising from a perceived mismatch between desired and actual social connections and from a lack of quality relationships whereby situations in which the intimacy one wishes for has not been realized (de Jong Gierveld, 1998). It represents a significant public health concern among older adults (Prohaska et al., 2020), with mounting evidence linking it to adverse outcomes, such as poorer physical health (Ong et al., 2016), cognitive decline (Boss et al., 2015), depression (Erzen and Çikrikci, 2018), worse quality of life (Beridze et al., 2020), and increased mortality risk (Holt-Lunstad et al., 2015). Notwithstanding the limited available research, loneliness seems also to affect more vulnerable populations such as people with dementia (PwD), who often report feeling lonelier than their typically aging counterparts (Holmén et al., 2000). Indeed, in cognitively declining people, despite efforts to maintain recognized social roles, the acknowledgement of their condition may result in others undermining their credibility, thereby precipitating social alienation and emotional detachment. Among PwD, loneliness has been linked to poorer general cognitive functioning (e.g., Moretti et al., 2024), more severe behavioural and psychological symptoms of dementia (BPSD), including delusions and hallucinations (Sun et al., 2021), as well as reduced functional abilities (e.g., Moretti et al., 2024) and lower quality of life (e.g., Carbone et al., 2022a).

Although loneliness is frequently treated as a unitary construct, a distinction has been proposed between social loneliness (the feeling of missing a social network that can provide a sense of companionship) and emotional loneliness (the perceived absence of close and intimate relationships; Weiss, 1975). This conceptual differentiation and the importance of considering these two loneliness components separately stem from the evidence that they have distinct implications for older adults’ health-related outcomes: Social loneliness has been related to a decline in cognitive functioning (particularly in executive functions; Schnittger et al., 2012) and reduced longevity whereas emotional loneliness has been linked to poor mental health, increased depressive symptoms, heightened risk of dementia, and all-cause mortality (O'Súilleabháin et al., 2019; Shibata et al., 2021; Tiikkainen and Heikkinen, 2005). Notably, among PwD, social loneliness has been linked to cognitive functioning, particularly language skills, as individuals experiencing loneliness may be less likely to engage in social interactions, with a negative impact on language abilities, and to dysphoric mood. Conversely, emotional loneliness seems more closely associated with quality of life, but this relationship may vary depending on the severity of dementia (Carbone et al., 2022a).

Another critical issue in understanding loneliness and its health-related effects lies in how it is conceptualized, either as a transient response to perceived social challenges (Zhaoyang et al., 2022) or as a relatively stable dispositional trait (Mund et al., 2019). The transient perspective views loneliness as a relational stressor arising from life events that disrupt or threaten social bonds (e.g., reduced social engagement, widowhood, institutionalization, or increased physical disability; see Aartsen and Jylhä, 2011; Jones et al., 1985). Accordingly, loneliness can be potentially modified and alleviated through interventions fostering social engagement and stimulating activities that strengthen social connections (Zhang et al., 2023). Differently, the dispositional perspective considers loneliness a stable individual difference, akin to a personality trait (Mund et al., 2019). Evidence from longitudinal meta-analyses in fact indicate that individual differences in terms of loneliness remain relatively stable across the majority of the lifespan (Graham et al., 2024; Mund et al., 2019), with some individuals more predisposed to experiencing loneliness regardless of their environment and life circumstances.

However, limited intervention studies have addressed loneliness in PwD (Cohen-Mansfield and Perach, 2015; see Szeto et al., 2025 for a recent meta-analysis). It has been shown that loneliness in older adults living in institutionalized care settings or nursing homes can be mitigated through activities that promote participation and enjoyment (Smith et al., 2023; Szeto et al., 2025). However, none of the available programs are explicitly designed for PwD (Szeto et al., 2025).

Cognitive Stimulation Therapy (CST), one of the most established and evidence-based psychosocial interventions for PwD, incorporates activities designed to promote participation and enjoyment within a structured approach (Desai et al., 2024; Woods et al., 2023). Specifically, CST involves engaging group activities that target multiple cognitive domains while fostering social connection in a respectful, person-centred context (Kitwood, 1997). This intervention has consistently shown benefits for cognitive functioning (e.g., global cognition, language), quality of life, depression and BPSD (Desai et al., 2024; Woods et al., 2023).

The cognitively and socially enriching environment CST promotes, therefore, may not only support cognition and behaviour but also help address psychosocial issues such as loneliness (Orfanos et al., 2021). However, only a few studies have been conducted to explore CST’s efficacy in ameliorating loneliness among PwD, and findings remain mixed. Atay and Bahadır Yılmaz (2025), using a unidimensional measure of loneliness, reported significant reductions following CST. In contrast, studies that distinguished between emotional and social loneliness yielded more nuanced results: Capotosto et al. (2017) observed improvements only in emotional loneliness in PwD, whereas Piras et al. (2017) did not find any significant changes in loneliness among individuals diagnosed with vascular dementia, a condition characterized by mood changes, including depression, which may explain the association between loneliness and increased risk of dementia of the vascular type (Sutin et al., 2023). On the other hand, no study has been conducted to investigate how loneliness, as a trait-like characteristic, might shape the effects of psychosocial interventions such as CST on established cognitive, psychological, behavioural functioning and quality of life outcomes among PwD.

To leverage the dual nature of loneliness, as a transient experience and a dispositional trait, alongside its distinct dimensions (social and emotional loneliness), the first aim of this study was therefore to further evaluate the efficacy of CST, compared to an active control group, in alleviating loneliness and its emotional and social dimensions among people with mild-to-moderate dementia.

Another aim of this study was to examine whether baseline levels of loneliness in the CST group could predict short- and long-term benefits in key outcomes typically examined in CST, including general cognitive functioning, language, mood, BPSD, and quality of life. Understanding such an issue could provide key insights into tailoring CST interventions, considering individual differences in perceived loneliness.

Based on previous evidence, we expected CST to lead to an overall reduction in loneliness (Atay and Bahadır Yılmaz, 2025), particularly emotional loneliness (Capotosto et al., 2017), by fostering reciprocal interactions in a supportive, non-judgmental environment where participants can feel emotionally connected. Given the lack of prior evidence on the long-term effects of CST on loneliness, we explored whether any short-term benefits in terms of ameliorated loneliness would be long lasting.

We also explored whether individual differences in baseline loneliness could predict the benefits of CST.

In particular, whether effects vary depending on the specific outcome considered, the loneliness constructs assessed (overall loneliness or its dimensions), and the assessment timepoints considered was explored. We can expect, in line with previous studies (Carbone et al., 2022a), baseline social loneliness to be related to benefits in cognitive and mood outcomes, whereas baseline emotional loneliness to be related to improvements in QoL. PwDs who experience heightened levels of loneliness were expected to derive more substantial advantages from the intervention than those with lower baseline levels of loneliness, thanks to the CST environment which reactivate and support their residual psychosocial skills (thus helping reduce social loneliness) and/or provide supportive contact and intimacy (thereby increasing emotional connectedness). At the same time, it could be also expected that PwD reporting lower baseline levels of loneliness, given their more preserved psychosocial resources (allowing them to establish social connections) and/or their ability to feel more easily embedded, profit more from CST than those with heightened levels of loneliness, as they can be more effectively engaged in CST sessions.

Because loneliness has been shown to affect PwD differently depending on the dementia stage (Carbone et al., 2022a; Holmén et al., 2000) and given that its associations with other outcomes may stem from depressive symptoms and the level of cognitive functioning rather than from perceived loneliness (Yu et al., 2016), baseline general cognitive functioning and depressive symptoms were included as control variables.

## Materials and methods

### Study design and participants

We selected data from individuals with dementia who participated in a previous single-blind, multicentre controlled clinical trial assessing the efficacy of the Italian adaptation of CST (see Carbone et al., 2021). Eligibility criteria (Spector et al., 2003) included: a diagnosis of major neurocognitive disorder according to the Diagnostic and Statistical Manual of Mental Disorder—fifth edition (American Psychiatric Association, 2013) in the mild to moderate range (Mini-Mental State Examination score ≥14); a Clinical Dementia Rating score of 1 or 2 (Hughes et al., 1982); adequate ability to understand and communicate; no neurodevelopmental disorders, premorbid intellectual disabilities, or acute physical illnesses that could interfere with participation; no severe behavioural symptoms that could hinder engagement; and no diagnosed comorbid psychiatric disorders.

For the present study, we included only participants who completed the de Jong Loneliness Scale assessing loneliness and its social and emotional dimensions, alongside measures of general cognition, language, mood, behaviour, and quality of life at pre-intervention, immediately after completing the treatment (post-intervention), and 3 months later (follow-up). This resulted in a final sample of 68 participants in the CST intervention group and 47 in the active control group. A total of 15 participants (7 from the intervention group and 8 from the control group) did not complete the follow-up assessment due to unpredictable reasons (e.g., health issues, discharge from the residential home, opting out).

### Measures

*Loneliness*. The *de Jong Gierveld Loneliness Scale* (de Jong Gierveld and Van Tilburg, 2010) is a questionnaire on perceived loneliness consisting of six items with responses on a five-point Likert scale. Three items assess social loneliness, and three items assess emotional loneliness. The dependent variables were the sum of the scores for total, social, and emotional loneliness, with higher scores corresponding to lower perceived loneliness.^1^

*Global cognitive functioning.* The *Mini-Mental State Examination* (MMSE; Folstein et al., 1975) covers five cognitive areas: temporal and spatial orientation, immediate and delayed verbal memory, language, attention, and praxis. The dependent variable was the sum of all items, with higher scores indicating better global cognitive functioning. The *Alzheimer’s Disease Assessment Scale—Cognitive subscale* (ADAS-Cog; Rosen et al., 1984) contains 11 tasks assessing orientation, memory, language, praxis, attention, and other cognitive abilities. The dependent variable was the total score, with higher scores indicating a more impaired cognitive functioning.

*Language*. The *Narrative Language Test* (NLT; Carlomagno et al., 2013) examines textual competence and discourse information content, assessing narrative abilities in terms of the effective communication of information. Participants are asked to describe a single figure and then sets of figures. Descriptions are recorded, transcribed verbatim, and segmented using correct information unit analysis, followed by a quantitative textual analysis. The dependent variable was the sum of the correctly and accurately reported items.

*Depressive and psychological and behavioural symptoms.* The *Cornell Depression Scale in Dementia* (CSDD; Alexopoulos et al., 1988) assesses depressive symptoms in PwD. It consists of 19 questions with responses on a three-point Likert scale. The dependent variable was the sum of scores from all items, with higher scores indicating greater severity of depressive symptoms. The *Neuropsychiatric Inventory* (NPI; Cummings et al., 1994) assesses the frequency and severity of 12 BPSD, including delusions, hallucinations, agitation/aggression, depression/dysphoria, anxiety, euphoria, apathy, disinhibition, irritability, aberrant motor behaviour, sleep disturbances, eating disorders. The dependent variable was the sum of the frequency * severity scores from all symptoms, with higher scores corresponding to greater severity and frequency of BPSD.

*Quality of life*. The *Quality of Life—Alzheimer’s Disease scale* (QoL-AD; Logsdon et al., 1999) includes 13 items assessing subjective components (e.g., perceived quality of life and psychological well-being) and objective components (e.g., behavioural competence and environment) of quality of life, rated on a four-point Likert scale. The dependent variable was the sum of all items, with higher scores indicating a better quality of life.

### Procedure

All participants attended 20 sessions over a period of 23 weeks.

Six were individual sessions, two for each of the pre-intervention, post-intervention and 3-months follow-up assessments conducted by trained psychologists who did not participate in the treatment program (see Carbone et al., 2021). During the first session, participants completed the MMSE and ADAS-Cog and in the second session, they were administered the de Jong Gierveld Loneliness Scale, NLT, and QoL-AD. The de Jong Gierveld Loneliness Scale and QoL-AD were rated by the participant in the presence of the experimenter, who provided assistance in case of difficulties in understanding the instructions and the items. The CSDD and NPI were completed by the care facility staff who has regular contact with the residents.

The remaining 14 sessions were group-based. During these sessions, the treatment group received the Italian adaptation (see Capotosto et al., 2017) of the original CST protocol developed by Spector et al. (2001, 2003). The 14 structured group sessions were delivered twice weekly over 7 weeks, in small groups of seven to eight participants. Each session followed a consistent structure: (i) a 10-min introduction, which included: a personalized welcome; choosing a group name and theme song; reviewing the day, month, year, weather, time, and the name and address of the residential center using a whiteboard; discussing current events and enjoying refreshments; (ii) 25-min main CST activities, covering different themes (e.g., sounds, food, categorizing objects, using money, word games) and tailored to the participants’ cognitive abilities (more difficult, for people with a MMSE of ≥19, or easier for people with a MMSE of 14–18); and (iii) a 10-min conclusion, thanking participants for their attendance and contributions, singing the theme song, reminding them of the time and content of the next session, and saying goodbye. The stimulation activities ensure that different cognitive domains (e.g., thinking, memory, problem-solving and language skills) are appropriately engaged according to participants interests and abilities, while the use of recurring activities (i.e., the warm-up, a song, the reality orientation board at the beginning, and the closing procedures) make sure there is continuity between sessions. Additionally, to augment the sense of togetherness and shared identity (Orfanos et al., 2021) that is instrumental in fostering the supportive and non-judgmental group atmosphere, the group’s name and song are defined during the first session and remain the same throughout the intervention. The CST program was delivered by two cofacilitators (one of them always a psychologist) who were members of the residential centre staff. Primary facilitators had prior experience in dementia care and group facilitation.

The active control group attended the same number of group sessions (twice weekly for 7 weeks) but engaged in standard educational activities offered by the residential care homes. These included reading and discussing articles from national and local newspapers or books, as well as creative activities such as coloring, painting, decorating, or cooking.

### Statistical analysis

Preliminarily, any differences between the CST and control groups at baseline were examined by means of Mann–Whitney *U* tests for each sociodemographic variable and outcome of interest.

To assess short-term benefits, linear mixed effect models were conducted for total, emotional and social loneliness, respectively, with Group (CST group vs. control group), Session (pre-intervention vs. post-intervention) and their interaction as predictors, baseline depressive symptoms (CSDD) and global cognitive functioning (MMSE) as covariates, and participant *id* as random factor. Since distributional assumptions were violated, a generalized mixed model with gamma distribution was employed. To interpret the Group * Session interactions, Bonferroni corrected post-hoc tests were conducted. The same analyses were also run considering the 3-months follow-up assessment. At this assessment phase, however, participant drop-out resulted in the loss of 7 participants in the CST group and 8 participants in the control group. To address missing data, a multiple imputation (MI) approach was employed for long-term analyses (see Supplementary Material). For ease of interpretation, the linear mixed models’ parameters were inverted so that positive coefficients would indicate an increase in loneliness and vice versa.

Next, to confirm the relationship between loneliness and the cognitive, behavioural and psychological outcomes of interest, Pearson correlations between baseline loneliness and the baseline scores of MMSE, ADAS-Cog, NLT, CSDD, NPI, and QoL-AD were calculated for the CST group only.

Finally, to investigate whether loneliness scores at baseline could predict changes in the cognitive, behavioural and psychological outcomes of interest, a series of linear models were conducted with MMSE, ADAS-Cog, NLT, CSDD, NPI, and QoL-AD short-term (post-intervention–pre-intervention) and long-term (follow-up–pre-intervention) gains, separately, as dependent variables, baseline emotional and social loneliness (or total loneliness only) as predictors, and pre-intervention scores for CSDD and MMSE as covariates. The same model was applied using either total loneliness, or its two dimensions, to compare explained variance in observed changes when a unitary conceptualization of loneliness or a dimensional approach were considered. Even in this case, a MI approach was employed for long-term analyses (see Supplementary Material). For ease of interpretation, the linear models’ parameters for loneliness were inverted so that positive coefficients would indicate that higher baseline loneliness was associated with greater gains and vice versa.

## Results

Table 1 shows the descriptive statistics of sociodemographics and outcomes of interest by group and assessment session. No significant differences between the two groups at baseline emerged.

**Table 1:** Descriptive statistics of socio-demographic characteristics and measures of interest by group (CST group and control group) and assessment session (pre-intervention, post-intervention, and 3-months follow-up) and results of groups comparisons at baseline.

	Baseline differences	CST group (*N* = 68; 46 F)			Active control group (*N* = 47; 29 F)		
		*M*	SD	Min–Max	*M*	SD	Min–Max
Age	*U* = 1,727; *p* = 0.46	81.67	7.48	61–94	82.64	7.44	62–94
Education	*U* = 1,418; *p* = 0.37	6.99	3.55	1–17	6.83	3.74	3–17

	Baseline differences	CST group (*N* = 68; 46 F)									Active control group (*N* = 47; 29 F)								
		Pre-intervention (*N* = 68)			Post-intervention (*N* = 68)			Follow-up (*N* = 61)			Pre-intervention (*N* = 47)			Post-intervention (*N* = 47)			Follow-up (*N* = 39)		
*M*	SD	Min–Max	*M*	SD	Min–Max	*M*	SD	Min–Max	*M*	SD	Min–Max	*M*	SD	Min–Max	*M*	SD	Min–Max
Total Loneliness	*U* = 1,509; *p* = 0.49	20.28	5.04	5–30	20.82	4.62	9–30	20.82	5.05	5–30	19.38	5.11	8–30	19.47	4.54	10–28	19.00	4.87	10–30
Emotional Loneliness	*U* = 1,581; *p* = 0.92	9.78	3.11	2–15	10.66	2.67	4–15	10.23	2.64	2–15	9.81	2.53	4–15	10.34	2.40	6–15	9.74	2.49	5–15
Social Loneliness	*U* = 1,344; *p* = 0.15	10.50	2.92	2–15	10.16	2.80	4–15	10.59	2.97	3–15	9.57	3.21	3–15	9.13	2.86	3–14	9.26	3.13	3–15
MMSE	*U* = 1,546; *p* = 0.76	20.15	4.08	13–28	20.63	4.36	9–29	20.70	4.33	11–28	19.88	4.05	10–27	19.30	3.93	11–28	19.46	3.79	11–26
ADAS-Cog	*U* = 1,716; *p* = 0.50	28.69	11.16	9–65	25.87	11.89	9–66	27.07	12.53	11–66	30.19	12.34	12–71	31.92	12.96	11–71	32.01	14.54	12–71
NLT	*U* = 1,482; *p* = 0.51	11.50	5.72	1–29	15.18	7.74	2–37	13.36	5.37	4–28	10.68	5.19	2–22	11.38	6.56	3–28	10.92	4.93	1–21
CSDD	*U* = 1,326; *p* = 0.12	5.04	5.11	0–20	3.01	3.24	0–11	4.36	4.99	0–18	3.62	4.43	0–23	4.13	5.55	0–22	4.95	5.94	0–26
NPI	*U* = 1,315; *p* = 0.10	11.44	12.74	0–46	7.50	9.48	0–45	13.26	16.04	0–58	7.94	11.43	0–48	10.23	19.70	0–96	11.75	22.21	0–104
QoL-AD	*U* = 1,354; *p* = 0.16	28.69	11.11	2–54	30.74	8.26	6–44	30.05	8.41	6–44	26.60	9.04	6–43	26.87	8.66	6–43	26.46	9.29	6–48

MMSE, Mini-Mental State Examination; ADAS-Cog, Alzheimer’s Disease Assessment Scale – Cognitive Subscale; NLT, Narrative Language Test; CSDD, Cornell Scale for Depression in Dementia; NPI, Neuropsychiatric Inventory; QoL-AD, Quality of Life - Alzheimer’s Disease; *U*, Mann–Whitney *U* test. Higher scores in the Loneliness Scale correspond to lower perceived total, social, emotional loneliness.

To verify the intervention efficacy on classical CST outcomes in this subsample, we conducted mixed-effects models for MMSE, ADAS-Cog, NLT, CSDD, NPI, and QoL-AD, assessing both short- and long-term benefits using Group (CST vs. control), Session (pre- vs. post-intervention or follow-up), their interaction as predictors, and participant *id* as random factor. CST confirms its short-term efficacy in improving the outcomes typically targeted by the intervention, with significant benefits observed in the CST group (see Supplementary Table S1). In the long term, positive effects were maintained for depressive symptoms (CSDD) (see Supplementary Table S2).

### Effect of CST on loneliness

For total loneliness, there was a significant main effect of Assessment Session, *B* = −0.73, *t* = −2.35, *p* = 0.02, with a decrease in total loneliness in both groups in the short-term. The main effect of Group, the interaction, and the covariates (CSDD and MMSE) were not significant (Table 2). At follow-up, there was a significant effect of the covariate CSDD, *B* = −0.26, *t* = −2.31, *p* = 0.02, with depressive symptoms predicting total loneliness. No significant main effects, interactions, or MMSE covariate effect were observed (Supplementary Table S3).

**Table 2:** Results from mixed-effect models for total, emotional, and social loneliness with group (CST vs. control), assessment session (pre-intervention vs. post-intervention) and their interactions as predictors and CSDD and MMSE baseline scores as covariate.

Effect	Short-term total loneliness (*R*^2^ = 0.116)			Short-term emotional loneliness (*R*^2^ = 0.120)			Short-term social loneliness (*R*^2^ = 0.157)		
	*B*	*t*	*p*	*B*	*t*	*p*	*B*	*t*	*p*
Group: CST vs. control (reference group)	−0.58	−0.54	0.59	−0.16	−0.26	0.80	−0.49	−0.80	0.43
Assessment session: post–pre (reference session)	**−0.73**	**−2.35**	**0.02**	**−1.10**	**−5.47**	**<0.001**	0.14	0.64	0.52
Group * assessment session	−0.53	−0.85	0.40	**−0.88**	**−2.18**	**0.03**	−0.14	−0.34	0.74
CSDD pre-intervention	−0.20	−1.87	0.06	−0.04	−0.67	0.51	**−0.16**	**−2.53**	**0.01**
MMSE pre-intervention	−0.08	−0.64	0.52	−0.07	−0.89	0.38	−0.01	−0.19	0.85

CSDD, Cornell Scale for Depression in Dementia; MMSE, Mini-Mental State Examination. Significant results in bold.

There were no significant main effects, interactions or MMSE covariate effect for social loneliness either in the short or long term (Tables 2 and Supplementary Table S3). However, depressive symptoms (CSDD) significantly contributed to explaining observed variance, *B* = −0.16, *t* = −2.53, *p* = 0.01 (pre- and post-intervention assessment) and *B* = −0.20, *t* = −2.89, *p* = 0.004 (pre-intervention–follow-up assessment).

As for emotional loneliness, there was a significant main effect of Assessment Session, *B* = −1.10, *t* = −5.47, *p* < 0.001, and a significant Group * Session interaction, *B* = −0.88, *t* = −2.18, *p* = 0.03. The CST group showed a greater decrease in emotional loneliness compared to the control group in the short term (post-intervention). The main effect of Group and covariates (CSDD and MMSE) were not significant (Table 2). When considering the follow-up assessment, no significant main effects, interactions, or covariates effect were found, suggesting that short-term benefits were no longer observable at follow-up (Supplementary Table S3).

### Effect of baseline loneliness in predicting short- and long-term benefits of CST

Focusing solely on the CST group, the correlations between baseline loneliness and baseline scores for the other outcomes of interest are presented in Table 3. Total loneliness at baseline was associated with all outcomes of interest. Emotional loneliness at baseline was found to be associated with general cognitive functioning measured by the ADAS-Cog, language, and quality of life. Social loneliness at baseline appeared to be associated with all outcomes of interest.

**Table 3:** Correlations between pre-intervention loneliness and pre-intervention in the investigated variables.

	Loneliness total pre-intervention	Emotional loneliness pre-intervention	Social loneliness pre-intervention
Pre-intervention MMSE	***r* = −0.299, *p* = 0.013**	*r* = −0.160, *p* = 0.194	***r* = −0.346, *p* = 0.004**
Pre-intervention ADAS-Cog	***r* = 0.381, *p* = 0.001**	***r* = 0.258, *p* = 0.033**	***r* = 0.382, *p* = 0.001**
Pre-intervention NLT	***r* = −0.474, *p* < 0.001**	***r* = −0.269, *p* = 0.026**	***r* = −0.530, *p* < 0.001**
Pre-intervention CSDD	***r* = −0.344, *p* = 0.004**	*r* = −0.141, *p* = 0.250	***r* = −0.443, *p* < 0.001**
Pre-intervention NPI	***r* = −0.306, *p* = 0.011**	*r* = −0.126, *p* = 0.307	***r* = −0.394, *p* < 0.001**
Pre-intervention QoL-AD	***r* = −0.591, *p* < 0.001**	***r* = −0.547, *p* < 0.001**	***r* = −0.436, *p* < 0.001**

Only CST group included (*N* = 68).

MMSE, Mini Mental State Examination; ADAS-Cog, Alzheimer’s Disease Assessment Scale – Cognitive Subscale; NLT, Narrative Language Test; CSDD, Cornell Scale for Depression in Dementia; NPI, Neuropsychiatric Inventory; QoL-AD, Quality of Life - Alzheimer’s Disease. Significant results in bold.

Regression analyses (see Table 4) showed no significant effects of baseline total loneliness, nor of covariates, for the MMSE, and the NLT in the short term.

**Table 4:** Results from linear models for short-term changes in measures of interest with total loneliness at pre-intervention as predictors and CSDD and MMSE at pre-intervention scores as covariate.

Effect	Short-term MMSE (*R*^2^ = 0.004)			Short-term ADAS-Cog (*R*^2^ = 0.031)			Short-term NLT (*R*^2^ = 0.097)			Short-term CSDD (*R*^2^ = 0.089)			Short-term NPI (*R*^2^ = 0.386)			Short-term QoL-AD (*R*^2^ = 0.214)		
	*B*	*t*	*p*	*B*	*t*	*p*	*B*	*t*	*p*	*B*	*t*	*p*	*B*	*t*	*p*	*B*	*t*	*p*
Total loneliness pre-intervention	−0.04	−0.54	0.59	−0.18	−1.24	0.22	−0.09	−0.88	0.39	**0.14**	**1.79**	**<0.001**	0.01	0.02	0.99	**0.58**	**3.06**	**0.003**
CSDD pre-intervention	−0.01	−0.14	0.89	**−0.29**	**−2.09**	**0.04**	0.16	1.54	0.13	–	–	–	**−0.97**	**−5.95**	**<0.001**	−0.28	−1.53	0.13
MMSE pre-intervention	–	–	–	−0.08	−0.47	0.64	0.11	0.13	0.39	−0.12	−1.21	0.23	−0.03	−0.14	0.89	0.24	1.07	0.29

Only CST group included (*N* = 68).

MMSE, Mini-Mental State Examination; ADAS-Cog, Alzheimer’s Disease Assessment Scale – Cognitive Subscale; NLT, Narrative Language Test; CSDD, Cornell Scale for Depression in Dementia; NPI, Neuropsychiatric Inventory; QoL-AD, Quality of Life in Alzheimer’s Disease Scale. Significant results in bold.

For the ADAS-Cog, there was no significant effect of baseline total loneliness. A significant effect of the covariate pre-intervention CSDD was found, *B* = −0.29, *t* = −2.09, *p* = 0.04, suggesting that higher depressive symptoms at baseline correlated with a greater improvement in global cognitive functioning. The covariate MMSE was not significant (Table 4).

As for CSDD, a significant effect of pre-intervention total loneliness was found, *B* = 0.14, *t* = 1.79, *p* < 0.001, suggesting that older adults who perceived less global loneliness at baseline experienced greater reduction in depressive symptoms (Table 4). The covariate MMSE was not significant (Table 4).

Regarding NPI, there were no significant effects of baseline total loneliness. A significant effect of the covariate pre-intervention CSDD was observed, *B* = −0.97, *t* = −5.95, *p* < 0.001, so that higher depressive symptoms at baseline correlated with a greater reduction in BPSD (Table 4).

For QoL-AD, a significant effect of pre-intervention total loneliness was observed, *B* = 0.58, *t* = 3.06, *p* < 0.001, suggesting that those PwD who felt lonelier -in general- at the start of the intervention showed a greater improvement in quality of life. The covariates MMSE and CSDD were not significant (Table 4).

As for long-term changes, no effects of baseline total loneliness, nor of covariates emerged for MMSE, ADAS-Cog, NLT, CSDD, NPI, and QoL-AD (Table 5).

**Table 5:** Results from linear models for short-term changes in measures of interest with social loneliness and emotional loneliness at pre-intervention as predictors and CSDD and MMSE at pre-intervention scores as covariate.

Effect	Short-term MMSE (*R*^2^ = 0.005)			Short-term ADAS-Cog (*R*^2^ = 0.018)			Short-term NLT (*R*^2^ = 0.115)			Short-term CSDD (*R*^2^ = 0.197)			Short-term NPI (*R*^2^ = 0.414)			Short-term QoL-AD (*R*^2^ = 0.324)		
	*B*	*t*	*p*	*B*	*t*	*p*	*B*	*t*	*p*	*B*	*t*	*p*	*B*	*t*	*p*	*B*	*t*	*p*
Social loneliness pre-intervention	−0.07	−0.51	0.62	−0.25	−0.89	0.38	−0.29	−1.41	0.16	**0.49**	**3.53**	**<0.001**	0.52	1.49	0.14	−0.34	−0.97	0.33
Emotional loneliness pre-intervention	−0.01	−0.11	0.91	−0.12	−0.52	0.60	0.06	0.34	0.73	−0.16	−1.28	0.21	−0.37	−1.35	0.18	**1.25**	**4.38**	**<0.001**
CSDD pre-intervention	−0.01	−0.21	0.83	**−0.30**	**−2.08**	**0.04**	0.13	1.20	0.24	–	–	–	**−0.88**	**−5.25**	**<0.001**	**−0.41**	**−2.35**	**0.02**
MMSE pre-intervention	–	–	–	−0.09	−0.50	0.62	0.09	0.68	0.50	−0.07	−0.71	0.48	0.02	0.10	0.92	0.14	0.65	0.52

Only CST group included.

MMSE, Mini-Mental State Examination; ADAS-Cog, Alzheimer’s Disease Assessment Scale–Cognitive Subscale; NLT, Narrative Language Test; CSDD, Cornell Scale for Depression in Dementia; NPI, Neuropsychiatric Inventory; QoL-AD, Quality of Life in Alzheimer’s Disease Scale. Significant results in bold.

Considering both loneliness dimensions, there were no significant effects of baseline social and emotional loneliness scores, nor of covariates, for the MMSE, and the NLT (Table 5).

For ADAS-Cog, there were no significant effects of baseline social and emotional loneliness. A significant effect of the covariate pre-intervention CSDD was found, *B* = −0.30, *t* = −2.08, *p* = 0.04, suggesting that having higher depressive symptoms at pre-intervention correlated with greater improvements in cognition after treatment. The covariate MMSE was not significant (Table 5).

As for CSDD, only a significant effect of pre-intervention social loneliness was found, *B* = 0.49, *t* = 3.53, *p* < 0.001, suggesting that older adults who perceived less social loneliness at baseline experienced greater reduction in depressive symptoms. The effects of emotional loneliness and the covariate MMSE were not significant (Table 5).

Regarding NPI, there were no significant effects of baseline social and emotional loneliness. A significant effect of the covariate pre-intervention CSDD was observed, *B* = −0.88, *t* = −5.25, *p* < 0.001 (Table 5). This indicates that higher depressive symptoms at the start of the intervention correlated with a greater reduction in BPSD.

For QoL-AD, a significant effect of pre-intervention emotional loneliness was observed, *B* = 1.25, *t* = 4.38, *p* < 0.001, suggesting that PwD who felt emotionally lonelier at baseline showed a greater improvement in quality of life (Table 5). The effect of social loneliness was not significant. A significant effect of the covariate pre-intervention CSDD was found, *B* = −0.41, *t* = −2.35, *p* = 0.02: with higher baseline depressive symptoms correlating with lower improvements in quality of life. The covariate MMSE was not significant (Table 5).

As for long-term changes (see Supplementary Table S5), no effects of baseline social and emotional loneliness scores, nor of covariates emerged for MMSE, ADAS-Cog, NLT, CSDD, and NPI. For QoL-AD, a significant effect of baseline emotional loneliness was found, *B* = 1.29, *t* = 3.73, *p* < 0.001, suggesting that PwD who felt emotionally lonelier at baseline showed a lower improvement in quality of life (see Supplementary Table S5). The effect of social loneliness and the covariates CSDD and MMSE were not significant (see Supplementary Table S5).

In all cases, the model incorporating both facets of loneliness explained more variance than the model using only total loneliness (see Supplementary Table S5).

## Discussion

This study represents a novel contribution to the growing body of research on loneliness in people with mild-to-moderate dementia, a population that has been generally underrepresented in loneliness studies despite their heightened vulnerability. Accounting for the transient and dispositional nature of loneliness and the importance of considering its social and emotional facets, we explored whether loneliness could be ameliorated by CST, one of the widely used evidence-based psychosocial interventions for PwD. We also investigated whether baseline levels of loneliness could predict short- and long-term benefits in key outcomes typically targeted by CST when the concurrent effects of depressive symptoms’ severity and baseline cognitive status were parcelled out.

Our results showed a nuanced efficacy of CST in reducing loneliness. No effect was found for total loneliness. Our findings therefore diverge from those of Atay and Bahadır Yılmaz (2025), who reported a significant reduction in loneliness following CST. This discrepancy may be attributed to differences in the measurement tools used. Whereas we employed the de Jong Gierveld Loneliness Scale, which distinguishes between emotional and social loneliness, Atay and Bahadır Yılmaz (2025) used a unidimensional measure of loneliness (i.e., UCLA Loneliness Scale–Short Form).

Though our results merit replication, the methodological difference in terms of questionnaires used underscores the importance of considering the multifaceted nature of loneliness when evaluating intervention outcomes.

When emotional loneliness was considered, in line with Capotosto et al. (2017), CST was found to significantly alleviate emotional loneliness in the short-term, compared to controls. In contrast, Piras et al. (2017) did not observe similar changes in this dimension; however, it is important to note that their sample included only individuals with vascular dementia—a subtype frequently associated with higher levels of apathy, anxiety, and depression (Kazui et al., 2016)—which may reduce responsiveness to interventions such as CST. The structured, supportive, and engaging environment of CST likely fosters emotional connections, providing a sense of attachment and intimacy that mitigates feelings of loneliness while acknowledging the participants’ emotional lives (Spector et al., 2001). Additionally, the intervention’s emphasis on meaningful participation may enhance overall psychological well-being, creating a positive environment where emotional connections naturally flourish, suggesting that CST benefits may emerge through the synergistic effects of its various components rather than through isolated improvements in specific domains (Woods et al., 2023).

Nevertheless, the limited duration of CST effects on emotional loneliness is a critical finding; in fact, benefits in this dimension did not persist at the 3-month follow-up. Several factors may explain this result. First, the time-limited nature of CST may not provide sufficient ongoing support to sustain the emotional connections formed during the intervention (Orrell et al., 2014). Once the sessions end, participants may struggle to maintain these connections, leading to a resurgence of emotional loneliness. Second, the progression of dementia may exacerbate feelings of loneliness over time, as cognitive and functional declines make it increasingly difficult for individuals to engage in meaningful social interactions (Hackett et al., 2019). Such a result is documented in CST studies showing that its benefits tend to decrease after the active intervention phase (Carbone et al., 2021; Orrell et al., 2014). Positive changes in emotional loneliness resulting from CST may therefore wane over time if not prompted by ongoing cognitive and social stimulation activities. Such a pattern of findings suggests the need to implement additional maintenance sessions to help reinforce these bonds and potentially sustain the short-term improvements over time, also when loneliness is concerned (Orrell et al., 2014; Sun et al., 2022). It is worth mentioning that except in a few studies (Carbone et al., 2021), mainly from out lab, CST’s long-term benefits have not been explored, therefore such an issue need to be further accounted more systematically.

When the social loneliness dimension was considered, consistent with prior research (Capotosto et al., 2017), no significant changes following CST were found. This suggests that although CST may effectively support emotional bonds and intimate connections in the group setting, it may fall short in addressing the broader sense of belonging and companionship. Social loneliness stems from a perceived lack of integration in a social network, and although CST provides opportunities for group interaction, it may not be sufficient to promote a deeper sense of social inclusion. This result could also be influenced by factors beyond the intervention, such as the availability of meaningful relationships in the nursing home, the quality of contact with family and friends outside the facility, and the individual’s sense of being part of a wider community, even in the institutional context (Eskimez et al., 2019). Indeed, several determinants help explain the feeling of social disconnectedness, such as the size and functioning of family relationships (particularly parent child bonds), nonkin relationships, gender, and health, which are not addressed (and cannot be ameliorated) by a psychosocial intervention (de Jong Gierveld and Peeters, 2003).

Interestingly, when we consider loneliness as a dispositional trait, our correlations confirmed that loneliness—emotional and social—is closely linked to cognitive functioning, quality of life, dysphoric mood, and BPSD, in line with previous findings (Carbone et al., 2022a; Sun et al., 2021).

The role of baseline loneliness in predicting CST’s outcomes provided mixed support to our hypotheses, while also offering valuable insights into how individual differences in loneliness shape intervention efficacy. Notably, our findings highlight that models incorporating both loneliness facets explained more variance than those using total loneliness alone, reinforcing the need for a multidimensional approach to understanding loneliness and its potential implications for intervention design.

Baseline loneliness did not significantly predict changes in global cognitive functioning (MMSE, ADAS-Cog), language (NLT), or BPSD (NPI), suggesting that these outcomes may be more influenced by other factors. Researchers are encouraged to further examine the impact of other individual characteristics, such as the severity of cognitive impairment or other individual factors such as personality, in predicting CST benefits in these specific and more general cognitive domains. This finding suggests that cognitive, linguistic, and behavioural changes derived from CST are mostly driven by the intervention direct cognitive stimulation rather than its psychosocial components (Woods et al., 2023).

However, individuals who reported lower social loneliness at baseline experienced greater reductions in depressive symptoms in the short-term. Such a pattern of findings is in line with the magnification hypothesis (Lövdén et al., 2012; see also Carbone et al., 2022b), which posits that individuals with greater baseline resources tend to benefit more from an intervention because their pre-existing characteristics provide a stronger foundation for amplifying potential benefits. In particular, feeling more connected to a group is likely to foster a sense of belonging and motivation, encouraging greater participation and reducing feelings of isolation, thereby alleviating depressive symptoms (Saleh et al., 2017). This result suggests that social loneliness, although not directly targeted by CST, plays a relevant role in shaping how individuals can respond to the intervention.

In contrast, individuals who reported higher emotional loneliness at baseline showed greater improvements in quality of life in the short- and long-term. This result is in line with the compensation hypothesis (Lövdén et al., 2012; see also Carbone et al., 2022b), which argues that individuals with lower baseline resources tend to benefit more from an intervention because they have more “room for improvement” as the treatment enables to recruit additional resources that help compensate for initial disadvantages. CST, with its emphasis on fostering supportive and reciprocal interactions, may have provided to those individuals feeling emotionally lonelier the affective closeness they were missing, significantly enhancing their quality of life. The sustained improvement in quality of life at follow-up further suggests that the emotional connections formed during CST, and probably consolidated in their everyday life, may have encouraged participants to seek out similar bonds even after the intervention ended. By addressing emotional loneliness, CST may have not only improved quality of life during the intervention but also empowered participants to continue building meaningful relationships in their daily lives. Such a speculation is consistent with research showing that older adults who lack close relationships often seek opportunities for intimacy (Fitzroy et al., 2022).

Notwithstanding these interesting results, the study has several limitations that should be acknowledged. In particular, reliance on a single measure of loneliness—the de Jong Gierveld Loneliness Scale—represents a limitation. Future research should therefore replicate and extend these findings using complementary measures that fully capture the complex nature of loneliness and its responsiveness to psychosocial interventions. At the same time, it is worth mentioning that the amount of variance explained by loneliness and its facets in CST outcomes remained limited, suggesting that additional factors likely contribute to the observed outcomes. As average levels of reported loneliness can vary widely across countries (Susanty et al., 2025), our findings should also be interpreted in light of the cultural context in which the study was conducted. Our sample was recruited in Italy, where family structures, social interactions, and the organization of dementia care may differ from those in other countries. These factors can shape both the subjective experience of loneliness and the consequent efficacy of psychosocial interventions such as CST (Aguirre and Werheid, 2017; Barreto et al., 2021) when individual differences are considered. Cross-cultural research is, thus, warranted to examine -specific or general- associations between loneliness and CST outcomes across diverse sociocultural environments.

Despite these limitations, our findings can have important practical implications for addressing loneliness in PwD. The significant short-term reduction in emotional loneliness highlights CST’s potential to address an impactful and detrimental aspect for this population. Importantly, implementing CST within routine clinical practice not only supports traditional outcomes typically targeted in psychosocial interventions for dementia—such as cognition, behavioural and psychological symptoms, and quality of life—but may also provide additional benefits by reducing loneliness, at least its emotional facet. This highlights CST’s potential as a multifaceted intervention that addresses both cognitive and psychosocial needs in PwD. Nonetheless, the lack of significant changes in social loneliness following CST implies that a more holistic approach—including complementary activities targeting the wider social isolation often experienced by PwD—should be considered.

Although loneliness can be considered a malleable state responsive to interventions, its stable, trait-like nature as an individual characteristic cannot be overlooked. This dual perspective was evident in the associations between baseline loneliness and changes brought about by CST, which varied depending on the specific facet of loneliness considered. From a practical standpoint, tailoring CST to the unique needs of individuals with varying levels of social and emotional loneliness may enhance its efficacy and guide clinicians in identifying those who are more responsive to treatment. CST structure and the activities proposed, valuing and promoting a strong sense of connection and belonging, would, therefore, contribute to alleviating depressive symptoms. Similarly, by providing support and encouraging group interactions, it can improve quality of life in those individuals whose affective closeness is fragile or compromised (i.e., people experiencing emotional loneliness).

From a clinical viewpoint, the CST interconnection of cognitive exercise and social elements may therefore be particularly valuable for PwD experiencing emotional and social loneliness prior to the intervention. Those individuals potentially socially withdrawn, lacking in self-confidence and likely unmotivated may, indeed, experience greater barriers to their optimal or potential functioning. These aspects will be possibly reduced by the intervention, as CST provides practice of both cognitive and social success in a cognitively, socially, and affective engaging environment. Ultimately, the CST person-centred approach, which emphasizes the inherent dignity and worth of individuals diagnosed with dementia, may prove to be especially advantageous for PwD. They are, in fact, likely confronting social and emotional isolation experiences intensified by social and personal stigma, which interfere with the person’s ability to participate in their community and maintain significant relationships. Conversely, the supportive and non-threatening environment of the CST can mitigate the internalized repercussions of social stigma, promoting self-efficacy, thereby preserving the individual’s capacity to engage in their community and uphold their interpersonal relationships.

In conclusion, this study is the first multicentre investigation showing that CST can also alleviate emotional loneliness, at least in the short-term, in individuals with dementia, highlighting intervention benefits extending beyond the traditionally examined outcomes, such as cognition. Moreover, these findings suggest the importance of considering individual differences in loneliness when implementing CST, for baseline levels of emotional and social loneliness may influence how individuals respond to the intervention, especially in specific domains. The role of baseline loneliness in predicting CST outcomes highlights the complex interplay between individual differences and intervention efficacy. Although social loneliness appears to amplify the benefits of CST in decreasing depressive symptoms (magnification effect), emotional loneliness contributes to improvements in quality of life (compensation effect) lasting in time. Addressing loneliness as a dispositional characteristic and counting it as a transient response to the context and environmental features would lead to therapeutic effect maximization in psychosocial interventions for PwD, such as CST.

## Data Availability

The raw data supporting the conclusions of this article will be made available by the authors, without undue reservation.

## Footnotes

^1^The 6-item version of the de Jong Gierveld Loneliness Scale, in the case of older adults living in long-term care facilities, is considered preferable to the 11-item version (Wong et al., 2022). As this study was part of a larger multicentre project in which numerous measures were administered (see Carbone et al., 2021), it also allowed to minimize participant burden and reduce fatigue. In addition, this short form has demonstrated good psychometric properties (de Jong Gierveld and Van Tilburg, 2006, 2010; Carbone et al., 2022a). In particular, in the present sample the scale showed a good-acceptable reliability (total loneliness: Cronbach’s *α* = 0.79; social loneliness subscale: Cronbach’s *α* = 0.83; emotional loneliness subscale: Cronbach’s *α* = 0.64).
