# Supplementary material for: Ameliorating loneliness through Cognitive Stimulation Therapy and the role of baseline loneliness in predicting cognitive, behavioural and psychological benefits in people with dementia
Source: Front Psychol. 2025 Oct 29;16:1656626. doi: 10.3389/fpsyg.2025.1656626 (PMC12605156; doi:10.3389/fpsyg.2025.1656626)
Supplement: Supplementary file 1 [file Supplementary_file_1.docx]

**Supplementary Materials**

**Ameliorating Loneliness through Cognitive Stimulation Therapy and the role of Initial Loneliness in Predicting cognitive, behavioural and psychological benefits in people with dementia**

**Effects of CST on typically-assessed outcomes**

To assess whether Cognitive stimulation therapy (CST) provided benefits in typically-assessed outcomes linear mixed effect models were conducted for Mini-Mental State Examination (MMSE), Alzheimer’s Disease Assessment Scale – Cognitive Subscale (ADAS-Cog), Narrative Language Test (NLT), Cornell Scale for Depression in Dementia (CSDD), Neuropsychiatric Inventory (NPI), Quality of Life in Alzheimer’s Disease Scale (QoL-AD) as dependent variables, with Group (CST group vs control group), Session (pre-intervention vs post-intervention) and their interaction as predictors and participant *id* as random factor. When distributional assumptions were violated, a generalized mixed model with gamma distribution was employed. To interpret the Group*Session interactions, Bonferroni corrected post-hoc tests were conducted. Results in Table S1 revealed a significant interaction for MMSE, B=1.08, t=2.29, p=0.02, indicating that global cognitive functioning was maintained in the CST group but declined in the control group post-intervention; no significant main effects emerged. For ADAS-Cog, a significant interaction was found, B=4.56, t=-4.62, p<0.001, showing improved cognitive functioning in the CST group and stability in the control group, with no main effects. Regarding the NLT, there was a significant main effect of Assessment Session, B=2.17, t=5.73, p<0.001, and a significant interaction, B=3.02, t=3.99, p<0.001, indicating increased linguistic abilities in the CST group and stability in the control group; no main effect of group was observed. A similar pattern was observed for CSDD, with a significant main effect of Assessment Session, B=-0.25, t=-2.36, p=0.02, and a significant interaction, B=-0.61, t=-2.83, p=0.005, indicating a reduction in depressive symptoms in the CST group only. No main effect of group emerged. Fo NPI, a significant interaction was observed, B=-6.28, t=-3.65, p<0.001, with a decrease in BPSD in the CST group and stability in the control group; no significant main effects were found. Finally, for QoL-AD, a significant main effect of Assessment Session, B=2.37, t=3.16, p=0.002, and a significant interaction, B=3.80, t=2.53, p=0.01, indicated an increase in perceived QoL in the CST group, while no change occurred in the control group. Again, no main effect of group was detected.

The same analyses were also run considering the follow-up assessment occurred 3 months after the treatment completion. To address missing data (7 participants in the CST group and 8 participants in the control group), a multiple imputation (MI) approach was employed for long-term analyses, creating several complete datasets where missing values were replaced with plausible values based on observed data. This method preserves the variability and uncertainty of missing data, ensuring robust statistical inferences. The same mixed models were applied to each dataset, and results were pooled using Rubin’s rules, which combine estimates and standard errors while accounting for variability within and between imputations, ultimately enhancing the reliability of the findings (Austin et al., 2021). Results in Table S2 indicated a significant interaction for CSDD, B=-2.31, t=-2.74, p=0.007, meaning that depressive symptoms remained stable in the CST group but increased in the control group at follow-up. Main effects were not significant. No significant main effects or interactions were found for MMSE, ADAS-Cog, NLT, NPI, or QoL-AD.

**Table S1**. Results from mixed-effect models for short-term changes in Mini-Mental State Examination (MMSE), Alzheimer’s Disease Assessment Scale – Cognitive Subscale (ADAS-Cog), and Narrative Language Test (NLT), Cornell Scale for Depression in Dementia (CSDD), Neuropsychiatric Inventory (NPI), and Quality of Life in Alzheimer’s Disease Scale (QoL-AD) with group (CST vs control), assessment session (pre-intervention vs post-intervention) and their interactions as predictors.

|  | **Short-term MMSE** | | | **Short-term ADAS-Cog** | | | **Short-term NLT** | | | **Short-term CSDD** | | | **Short-term NPI** | | | **Short-term QoL-AD** | | |
| --- | --- | --- | --- | --- | --- | --- | --- | --- | --- | --- | --- | --- | --- | --- | --- | --- | --- | --- |
| **Effect** | ***B*** | ***t*** | ***p*** | ***B*** | ***t*** | ***p*** | ***B*** | ***t*** | ***p*** | ***B*** | ***t*** | ***p*** | ***B*** | ***t*** | ***p*** | ***B*** | ***t*** | ***p*** |
| Group: CST vs control (reference group) | .74 | 1.01 | .32 | -3.59 | -1.63 | .11 | 2.09 | 1.79 | .08 | .33 | .42 | .68 | .53 | .22 | .82 | 2.50 | 1.18 | .24 |
| Assessment Session: post – pre (reference session) | -.05 | -.23 | .82 | -.53 | -1.08 | .28 | **2.17** | **5.73** | **<.001** | **-.25** | **2.36** | **.02** | -.80 | -.93 | .35 | **2.37** | **3.16** | **.002** |
| Group*Assessment Session | **1.08** | **2.29** | **.02** | **-4.56** | **-4.62** | **<.001** | **3.02** | **3.99** | **<.001** | **-.61** | **-2.83** | **.005** | **-6.28** | **-3.65** | **<.001** | **3.80** | **2.53** | **.01** |

**Note**: *MMSE = Mini-Mental State Examination; ADAS-Cog = Alzheimer’s Disease Assessment Scale – Cognitive Subscale; NLT = Narrative Language Test; CSDD = Cornell Scale for Depression in Dementia; NPI = Neuropsychiatric Inventory; QoL-AD = Quality of Life in Alzheimer’s Disease Scale. Significant results in bold.*

|  | **Long-term MMSE** | | | **Long-term ADAS-Cog** | | | **Long-term NLT** | | | **Long-term CSDD** | | | **Long-term NPI** | | | **Long-term QoL-AD** | | |
| --- | --- | --- | --- | --- | --- | --- | --- | --- | --- | --- | --- | --- | --- | --- | --- | --- | --- | --- |
| **Effect** | ***B*** | ***t*** | ***p*** | ***B*** | ***t*** | ***p*** | ***B*** | ***t*** | ***p*** | ***B*** | ***t*** | ***p*** | ***B*** | ***t*** | ***p*** | ***B*** | ***t*** | ***p*** |
| Group: CST vs control (reference group) | .91 | 1.05 | .30 | -2.85 | -1.11 | .27 | 1.30 | 1.33 | .19 | .51 | .53 | .60 | 2.97 | 1.06 | .29 | 3.17 | 1.73 | .87 |
| Assessment Session: follow-up – pre (reference session) | -.44 | -.95 | .36 | 1.32 | .65 | .54 | .57 | .94 | .36 | .43 | .50 | .64 | 3.64 | 1.81 | .92 | -.60 | -.51 | .62 |
| Group*Assessment Session | 1.12 | 1.35 | .19 | -.76 | -1.19 | .26 | 1.63 | 1.56 | .12 | **-2.31** | **-2.74** | **.007** | -2.41 | -.83 | .41 | 1.66 | .85 | .40 |

**Table S2**. Multiple Imputation results from mixed-effect models for long-term changes in MMSE, ADAS-Cog, NLT, CSDD, NPI, and QoL-AD with group (CST vs control), assessment session (pre-intervention vs post-intervention) and their interactions as predictors.

**Note**: *MMSE = Mini-Mental State Examination; ADAS-Cog = Alzheimer’s Disease Assessment Scale – Cognitive Subscale; NLT = Narrative Language Test; CSDD = Cornell Scale for Depression in Dementia; NPI = Neuropsychiatric Inventory; QoL-AD = Quality of Life in Alzheimer’s Disease Scale. Significant results in bold.*

**Long-term effects of CST on loneliness**

To assess whether CST provided long-term benefits in loneliness, linear mixed effect models were conducted for total, emotional and social loneliness, respectively, with Group (CST group vs control group), Session (pre-intervention vs post-intervention) and their interaction as predictors, baseline depressive symptoms (CSDD) and global cognitive functioning (MMSE) as covariates, and participant *id* as random factor. Since distributional assumptions were violated, a generalized mixed model with gamma distribution was employed. To interpret the Group*Session interactions, Bonferroni corrected post-hoc tests were conducted. At follow-up, however, participant drop-out resulted in the loss of 7 participants in the CST group and 8 participants in the control group. To address missing data, a MI approach was employed for long-term analyses. For ease of interpretation, the linear mixed models’ parameters were inverted so that positive coefficients would indicate an increase in loneliness and vice versa. Results in Table S3 showed that no significant main effects, interactions, influences were observed, suggesting that short-term benefits faded at follow-up. However, a significant effect of the covariate CSDD emerged for both total loneliness, B=-0.26, t=-2.31, p=0.02, and social loneliness, B=-0.20, t=-2.89, p=0.004, suggesting that depressive symptoms significantly contributed to explaining variance in general and social loneliness. No other covariates showed significant effects.

**Table S3**. Multiple Imputation results from mixed-effect models for emotional and social loneliness with group (CST vs control), assessment session (pre-intervention vs follow-up) and their interactions as predictors and CSDD and MMSE baseline scores as covariate.

|  | **Long-term Total Loneliness**  **(R^2^ = .176)** | | | **Long-term Emotional Loneliness (R^2^ = .089)** | | | **Long-term Social Loneliness (R^2^ = .254)** | | |
| --- | --- | --- | --- | --- | --- | --- | --- | --- | --- |
| **Effect** | ***B*** | ***t*** | ***p*** | ***B*** | ***t*** | ***p*** | ***B*** | ***t*** | ***p*** |
| Group: CST vs control (reference group) | -.77 | -.70 | .49 | .01 | .02 | .98 | -.78 | -1.07 | .29 |
| Assessment Session: Follow-up – pre (reference session) | -.32 | -.70 | .49 | -.53 | -1.82 | .07 | .25 | .70 | .49 |
| Group*Assessment Session | -.75 | -.86 | .39 | -.82 | -1.53 | .13 | -.54 | -.77 | .45 |
| CSDD pre-intervention | **-.26** | **-2.31** | **.02** | -.06 | -1.00 | .33 | **-.20** | **-2.89** | **.004** |
| MMSE pre-intervention | -.08 | -.52 | .61 | -.04 | -.62 | .54 | -.03 | -.26 | .80 |

**Note**: *CSDD = Cornell Scale for Depression in Dementia; MMSE = Mini-Mental State Examination. Significant results in bold.*

**Effects of baseline loneliness in predicting long-term benefits of CST**

To investigate whether loneliness scores at baseline could predict changes in the cognitive, behavioural and psychological outcomes of interest, a series of linear models were conducted with MMSE, ADAS-Cog, NLT, CSDD, NPI, and QoL-AD long-term (follow-up - pre-intervention) gains as dependent variables, baseline emotional and social loneliness as predictors, and pre-intervention scores for CSDD and MMSE as covariates. The same model was applied using either only total loneliness, or its two facets, to compare whether a unitary conceptualization of loneliness or a dimensional approach explained more variance in the observed changes. During this assessment phase, drop-out led to the loss of 7 participants. For this reason, a MI approach was employed for long-term analyses. Again, for ease of interpretation, the linear models’ parameters for loneliness were inverted so that positive coefficients would indicate that higher baseline loneliness was associated with greater gains and vice versa Results in Table S4 showed that no effects of baseline total loneliness, nor of covariates emerged for MMSE (R^2^=0.044), ADAS-Cog (R^2^=0.019), NLT (R^2^=0.080), CSDD (R^2^=0.193), NPI (R^2^=0.020), and QoL-AD (R^2^=0.146). Considering the two facets, Table S5 reported that no effects of baseline social and emotional loneliness scores, nor of covariates emerged for MMSE (R^2^=0.060), ADAS-Cog (R^2^=0.048), NLT (R^2^=0.085), CSDD (R^2^=0.212), and NPI (R^2^=0.071) (Table S5). For QoL-AD, a significant effect of baseline emotional loneliness was found, B=1.29, t=3.73, p<0.001, suggesting that PwD who felt less emotionally lonely at the start of the intervention showed a lower improvement in QoL (R^2^=0.255). The effect of social loneliness and the covariates CSDD and MMSE were not significant.

**Table S4**. Multiple Imputation results from linear models for long-term changes in measures of interest with social loneliness and emotional loneliness at pre-intervention as predictors and CSDD and MMSE at pre-intervention scores as covariate. Only CST group included.

|  | **Long-term MMSE (R^2^ = .044)** | | | **Long-term ADAS-Cog (R^2^ = .019)** | | | **Long-term NLT (R^2^ = .080)** | | | **Long-term CSDD (R^2^ = .193)** | | | **Long-term NPI (R^2^ = .020)** | | | **Long-term QoL-AD (R^2^ = .146)** | | |
| --- | --- | --- | --- | --- | --- | --- | --- | --- | --- | --- | --- | --- | --- | --- | --- | --- | --- | --- |
| **Effect** | ***B*** | ***t*** | ***p*** | ***B*** | ***t*** | ***p*** | ***B*** | ***t*** | ***p*** | ***B*** | ***t*** | ***p*** | ***B*** | ***t*** | ***p*** | ***B*** | ***t*** | ***p*** |
| Total loneliness pre-intervention | -.11 | -1.34 | .20 | -.07 | -.36 | .72 | .12 | .89 | .38 | .01 | .02 | .98 | .03 | .09 | .93 | .57 | 2.08 | .05 |
| CSDD pre-intervention | -.03 | -.39 | .70 | -.13 | -.67 | .51 | -.19 | -1.57 | .12 | - | - | - | -.18 | -.57 | .57 | -.14 | -.66 | .51 |
| MMSE pre-intervention | - | - | - | -.34 | -1.55 | .13 | -.01 | -.05 | .96 | -.05 | -.40 | .69 | -.41 | -.84 | .41 | .03 | .09 | .93 |

**Note**: *MMSE = Mini-Mental State Examination; ADAS-Cog = Alzheimer’s Disease Assessment Scale – Cognitive Subscale; NLT = Narrative Language Test; CSDD = Cornell Scale for Depression in Dementia; NPI = Neuropsychiatric Inventory; QoL-AD = Quality of Life in Alzheimer’s Disease Scale. Significant results in bold.*

**Table S5**. Multiple Imputation results from linear models for long-term changes in measures of interest with social loneliness and emotional loneliness at pre-intervention as predictors and CSDD and MMSE at pre-intervention scores as covariate. Only CST group included.

|  | **Long-term MMSE (R^2^ = .060)** | | | **Long-term ADAS-Cog (R^2^ = .048)** | | | **Long-term NLT (R^2^ = .085)** | | | **Long-term CSDD (R^2^ = .212)** | | | **Long-term NPI (R^2^ = .071)** | | | **Long-term QoL-AD (R^2^ = .255)** | | |
| --- | --- | --- | --- | --- | --- | --- | --- | --- | --- | --- | --- | --- | --- | --- | --- | --- | --- | --- |
| **Effect** | ***B*** | ***t*** | ***p*** | ***B*** | ***t*** | ***p*** | ***B*** | ***t*** | ***p*** | ***B*** | ***t*** | ***p*** | ***B*** | ***t*** | ***p*** | ***B*** | ***t*** | ***p*** |
| Social loneliness pre-intervention | -.23 | -1.31 | .21 | .28 | .77 | .45 | .23 | .89 | .38 | .31 | 1.68 | .10 | .93 | 1.33 | .19 | -.44 | -.87 | .40 |
| Emotional loneliness pre-intervention | -.02 | -.19 | .85 | -.32 | -1.12 | .27 | .04 | .20 | .85 | -.26 | -1.61 | .12 | -.62 | -1.27 | .21 | **1.29** | **3.73** | **<.001** |
| CSDD pre-intervention | -.05 | -.63 | .53 | -.07 | -.36 | .72 | -.17 | -1.36 | .18 | - | - | - | -.01 | -.03 | .97 | -.29 | -1.33 | .19 |
| MMSE pre-intervention | - | - | - | -.30 | -1.37 | .18 | .01 | .04 | .97 | -.01 | -.06 | .95 | -.33 | -.69 | .50 | -.10 | -.27 | .79 |

**Note**: *MMSE = Mini-Mental State Examination; ADAS-Cog = Alzheimer’s Disease Assessment Scale – Cognitive Subscale; NLT = Narrative Language Test; CSDD = Cornell Scale for Depression in Dementia; NPI = Neuropsychiatric Inventory; QoL-AD = Quality of Life in Alzheimer’s Disease Scale. Significant results in bold.*

**References**

Austin, P. C., White, I. R., Lee, D. S., & van Buuren, S. (2021). Missing data in clinical research: a tutorial on multiple imputation. *Canadian Journal of Cardiology*, *37*(9), 1322-1331.
